# Interface failure modes explain non-monotonic size-dependent mechanical properties in bioinspired nanolaminates

**DOI:** 10.1038/srep23724

**Published:** 2016-03-31

**Authors:** Z. Q. Song, Y. Ni, L. M. Peng, H. Y. Liang, L. H. He

**Affiliations:** 1 CAS Key Laboratory of Mechanical Behavior and Design of Materials,University of Science and Technology of China, Hefei, Anhui 230026, P. R. China

## Abstract

Bioinspired discontinuous nanolaminate design becomes an efficient way to mitigate the strength-ductility tradeoff in brittle materials via arresting the crack at the interface followed by controllable interface failure. The analytical solution and numerical simulation based on the nonlinear shear-lag model indicates that propagation of the interface failure can be unstable or stable when the interfacial shear stress between laminae is uniform or highly localized, respectively. A dimensionless key parameter defined by the ratio of two characteristic lengths governs the transition between the two interface-failure modes, which can explain the non-monotonic size-dependent mechanical properties observed in various laminate composites.

Laminated composite architectures have been widely used to make tough and damage tolerant materials which are intrinsically brittle^1^,^2^,^3^,^4^. It is well accepted that interface failure by delamination or debonding and/or friction between adjacent laminae plays an important role in enhancing toughness and damage tolerance^5^,^6^,^7^,^8^,^9^,^10^,^11^. Usually laminated composites are continuous, i.e. ceramic-metal laminates^12^,^13^, metal-intermetallic multilayers^14^,^15^, and multilayered ceramics with weak interlayer^1^,^16^,^17^,^18^ etc. When the strength of the weak interlayer bond is below a critical value compared to the strength of the lamina, the nucleated cracks in each lamina are deflected onto the interface followed by interface failure^5^,^6^,^7^, then it becomes a discontinuously laminated structure. Within the structure, the penetrations of these cracks into adjacent discontinuous laminae that usually lead to a straight propagation path and a catastrophic failure are significantly mitigated. Instead, a non-catastrophic fracture called as a “graceful failure” with the highly meandering crack path is observed^1^,^15^,^16^,^17^,^18^,^19^. Biological materials, i.e. nacre and bone are such good examples to achieve excellent mechanical performance by controlling interface failure via delicate hybrid discontinuous laminate design, a so called brick-and-mortar microstructure^20^,^21^ which is the incorporation of inorganic platelets with optimal aspect ratio and nanometers thick organic weak interlayer. Therefore, fabrication of biomimetic discontinuously laminated composites opens a new way towards superb mechanical properties^21^,^22^,^23^,^24^.

It is known that weak interface can arrest the cracks in the laminae, deflect them onto the interface and facilitate interface failure. The weak interlayer is unnecessary to be soft organic component^25^. Recent studies further show that there are significant size-dependent mechanical properties in discontinuously laminated composites under uniaxial stretch. They are definitely related to the interface failure since the unique stress transfer mechanism in these composites is the lamina/platelet in tension and the interface in shear. Once the maximum shear stress at the edge of the laminae is beyond the interface strength the interface starts to fail and the failure zone can propagate through the overlapping domain which varies with the platelet size and the platelet arrangement^26^,^27^,^28^,^29^,^30^,^31^,^32^,^33^,^34^,^35^,^36^. Theoretic analysis indicates that the aspect ratio of the platelet in nacre has a characteristic value, at which the platelet and the interface simultaneously undergo uniform failure and that is why nacre exhibits optimal mechanical properties^26^,^37^,^38^,^39^,^40^. In contrast, another experimental results report that the polymer/nanoclay nacre-mimetics exhibit high toughness and interface failure with low aspect ratio nanoplatelets, while they exhibit superior stiffness and strength with large aspect ratio nanoplatelets, in accompany with the failure mode changing from interface failure to platelet fracture^33^. The failure mode transition depends on where the maximum stress first reaches the failure point. The effects of the interfacial elasto-plasticity and the overlapping length on the stress field developed in the laminated structure are identified^34^. More interestingly, a non-monotonic size-dependency of the mechanical response is observed in the case of even no platelet fracture. The stiffness, strength, failure strain and toughness can be synergistically increased with the increase of the overlapping length in discontinuously overlapped ply carbon/epoxy composites, and they show a ductile behavior wherein the interface fails progressively although their components are brittle^35^. However in 3D-printed laminated composites under uniaxial stretch the measured failure strain is found to decrease with the increase of the overlapping length, and they exhibit a brittle behavior wherein the propagation of the interface failure is catastrophic^36^. These experimental data seems contradictory. A unified explanation about the size effect of the interface failure in such structures is needed. In most previous theoretic efforts based on interfacial fracture mechanics, the interface failure is considered as a crack. Although when the crack deflects onto the interface has been studied extensively, it remains unclear how the interface failure proceeds and how its propagation correlates the size-dependent mechanical behavior, as well as the so called “graceful failure” with enhanced work of failure.

In this paper, we aim to theoretically elucidate the size effect of the interface failure in such structures mentioned in the literatures. We use a shear lag model to calculate the stress field in the discontinuously laminated composite^34^,^41^,^42^,^43^,^44^ under uniaxial stretch. The interface failure zone is considered as a debonding zone wherein the shear stress drops to zero when the failure criterion is met. We then adopt a stress-based criterion to characterize the condition of the interface failure^5^ instead of the energy-based criterion usually used in the interfacial fracture mechanics^6^. The consistency of the both criterions is identified^45^. In fact the shear lag model with the stress-based failure criterion shows its capability to study the interface failure in layered nanoceramic composites compared with molecular modeling^46^. Our analytical results for the regularly staggered laminated structure show that the non-monotonic size effect of the interface failure is attributed to the propagation mode transition of the interface failure zone, which is a function of the overlapping length. At a small overlapping length, the interface exhibits catastrophic failure corresponding to the brittle feature, while it shows a steady and progressive failure above a critical overlapping length. The size-dependent mechanical response in the randomly staggered laminates is also discussed. The staggering randomness is found to suppress the single-sided interface failure on the interface with short overlapping length and to promote the double-sided interface failure which significantly increases the failure strain and the work of failure. The calculated step-like stress-strain response that exhibits the feature of “graceful failure” in these randomly staggered laminates is obtained. Its formation can be roughly explained as the result of superposition of propagation of interface failure involving different overlapping lengths.

## Results

### Mechanical response in regularly staggered laminates

**Figure 1:**
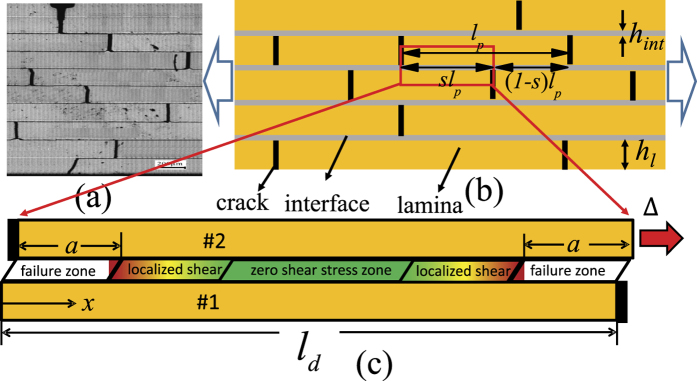
Schematic illustrating (**a**) the image adapted from ref. 47 for discontinuous Al_2_O_3_/LaPO_4_ laminates after cracking in each layer, (**b**) sketch of the discontinuously laminated structure for modeling, (**c**) the shear-lag model for the interface failure process.

We consider the interface failure process of a typical discontinuous laminate under uniaxial stretch *ε*_*c*_ as shown in Fig. 1. The discontinuous laminate can be a continuous laminate after the crack propagation in each lamina is arrested at the interface between lamina (see Fig. 1(a)) or inorganic platelets bonded by weak interlayer with nacre-like brick-and-mortar structure. The representative volume element (RVE) of such structure depends on the size of the platelets *l*_*p*_ and their arrangements. When the arrangement is regularly staggered with the offset *s* = 0.5 (Fig. 1(b)), the two-dimensional RVE is simplified as shown in Fig. 1(c) and a shear-lag model can be analytically solved to obtain the stress field in the structure. While if the arrangement is randomly staggered, the RVE becomes complex, and the shear-lag model is numerically solved via an overdamping relaxation method to obtain the stress field^34^. In the nonlinear shear-lag model, the interface is assumed to be elastic with *τ* = *G*_int_*γ* linking the shear stress *τ* and strain *γ* by the shear modulus *G*_int_ and it undergoes brittle failure after the shear stress is above its shear strength *τ*^*f*^ with the failure strain 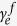 for simplicity. The mechanical equilibrium is governed by 
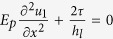
 for the platelet #1 and 
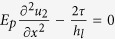
 for the platelet #2 with *E*_*p*_, *h*_*l*_, *u*_1_, *u*_2_ the Young’s modulus, the thickness of the platelet and the displacement in the platelets #1 and #2, respectively. After the stress fields are solved (see the details in the Supplementary Material Appendix A), the average stress in the RVE has the form


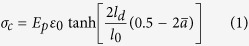


where 
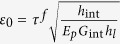
 is a characteristic strain with *h*_int_ the thicknesses of the interlayer, *l*_*d*_ is the overlapping length defined in Fig. 1(c), and 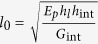 is a critical length that characterizes the distribution of the shear stress on the interface, i.e. the shear stress is almost uniform as *l*_*d*_ ≤ 0.5*l*_0_. 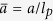 is the dimensionless length of the interface failure zone. At a given uniaxial stretch *ε*_*c*_ the value of 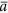 is determined by the following equation


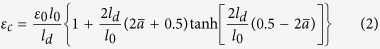


**Figure 2:**
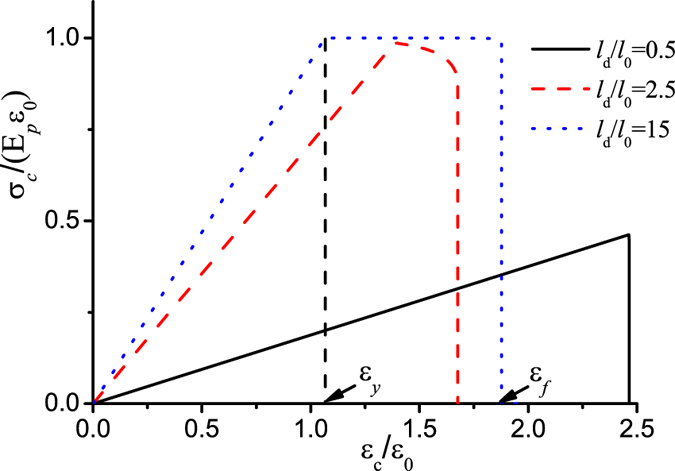
The calculated stress-strain curve under different overlapping length in regularly staggered discontinuous laminates.

Figure 2 plots the calculated effective stress-strain curve of the RVE in Fig. 1(c) under different values of *l*_*d*_ by substituting the value of 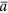 in eq. (2) into eq. (1). The result demonstrates a size-dependent mechanical response. When the overlapping length is smaller than a critical value, the failure of the structure is brittle, however it exhibits a ductile behavior after the overlapping length is larger than a critical value. The yield strain *ε*_*y*_ is defined at which interface failure starts to appear, namely, the value of 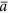 changes from zero to nonzero.


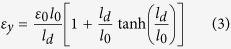


The failure strain of the RVE 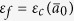 with 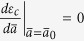 is defined as the maximum attainable applied stretch before the complete failure which poses an energetic instability. We noted that this criterion is consistent with the elastic energetic instability under the strain-controlled boundary condition since 
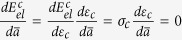
 in the case of *σ*_*c*_ > 0 is the same as 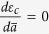.

**Figure 3:**
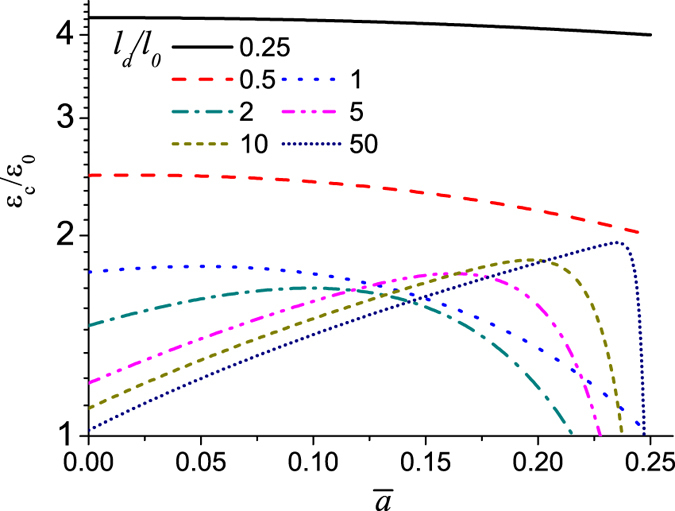
Plot of the applied stretch as a function of the size of the interface failure zone at different values of *l*_*d*_/*l*_0_.

**Figure 4:**
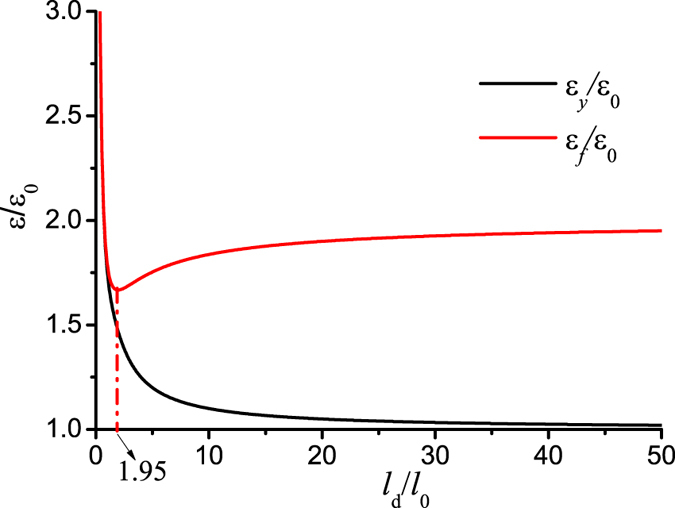
Plots of the yield and failure strains as a function of *l*_*d*_/*l*_0_ in regularly staggered discontinuous laminates.

**Figure 5:**
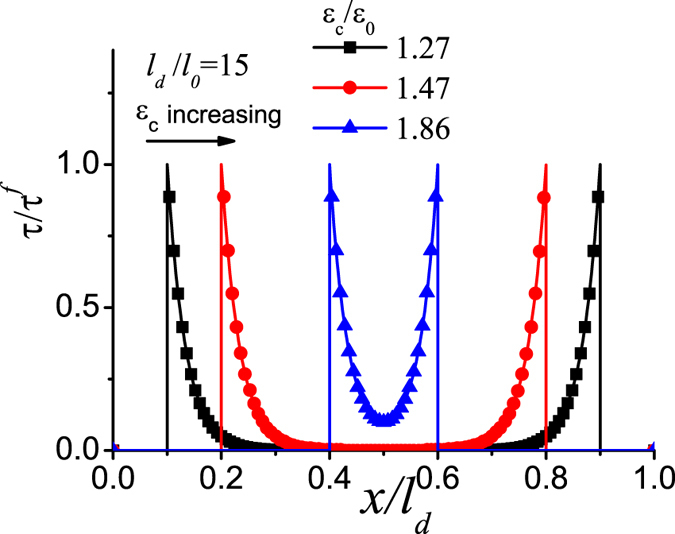
The sequential profile of the localized shear stress at the interface with the increase of the applied strain at *l*_*d*_/*l*_0_ = 15.

Figure 3 plots the applied stretch as a function of the size of the interface failure zone at different values of the dimensionless key parameter *l*_*d*_/*l*_0_. It shows that when *l*_*d*_/*l*_0_ is small, the maximum applied stretch reaches at 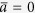 and this implies that once the interface failure occurs its propagation is catastrophic, while if *l*_*d*_/*l*_0_ is sufficiently large, the maximum applied stretch reaches at 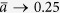, and this signifies that there is a steady and progressive interface failure process. Figure 4 shows that the yield strain monotonically decreases till the value of *ε*_0_ with the increase of *l*_*d*_/*l*_0_, however, the failure strain firstly decreases, then increases and finally converges toward 2*ε*_0_ at sufficiently large value of *l*_*d*_/*l*_0_. The result in Fig. 4 indicates that on the one hand, the failure strain could be much larger than the yield strain where a progressive non-catastrophic interface failure contributes a ductile behavior in the laminate although the platelets and the interlayer are intrinsically brittle, on the other hand, in the structure with *l*_*d*_/*l*_0_ < 1.95 the failure strain is very close to the yield strain, and it exhibits a brittle behavior. The reason is that the shear stress on the interface is almost uniform and the interface tends to undergo uniform rupture. In the case of large *l*_*d*_/*l*_0_, the shear stress is highly localized. Figure 5 plots the shear stress profile along the interface with the increase of uniaxial stretch at *l*_*d*_/*l*_0_ = 15. The result clearly shows that the interface is separated into three distinct zones: failure zone, localized shear zone, and zero shear stress zone. With the increase of the uniaxial stretch, the failure zone can grow steadily without the drop of the tensile stress in the platelets at the expense of decreasing the zero shear stress zone until it disappears. Therefore it is the steady profile of the localized shear stress that guarantees a progressive interface failure. Our simple model thus elucidates two distinct interface failure modes: the catastrophic and non-catastrophic interface failures. The former occurs when the shear stress on the interface is uniform and uniform interface failure is favorable. The latter takes place when the shear stress on the interface is localized and progressive interface failure dominates. Control of the interface mode depends on the value of *l*_*d*_/*l*_0_ which determines the distribution of the interfacial shear stress. The size-dependent interface failure mode can explain why the failure strain in the 3D-printed laminate^36^ decreases while the failure strain in the discontinuously overlapped ply carbon/epoxy laminate^35^ increases with the increase of the overlapping length. Based on estimating the value of *l*_*d*_/*l*_0_ in the two laminates^35^,^36^, we find that *l*_*d*_/*l*_0_ < 2 (*l*_0_ = 5/*λ* with the interface plasticity taken into account^34^,^36^, *l*_*d*_ = *L*, *l*_*d*_/*l*_0_ ≈ *λL*/5) in the former while *l*_*d*_/*l*_0_ ≈ 21 (*l*_0_ = 37 *mm*, *l*_0_ = 7.64 *mm*) in the latter^35^. From Fig. 4, the two laminates are exactly located the two sides of the deflection point *l*_*d*_/*l*_0_ ≈ 2, respectively. Therefore their failure strains show different size-dependency.

### Mechanical response in randomly staggered laminates

The overlapping length depends on not only the size of the platelets but also their arrangements. In the real case as shown in Fig. 1(a), the discontinuous laminates are more or less randomly staggered. Hence the overlapping length is not uniform any more but widely distributed. We need to discuss the effect of the staggering randomness on the size-dependent mechanical response. We introduce a distributed offset *s* to characterize the staggered arrangement. For example, the random staggering can be generated by a normal distribution with the mean 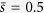 and a standard deviation Δ*s*. In this setting, the average overlapping length 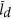 is equal to 0.5*l*_*p*_, the large value of Δ*s* implies the overlapping length is very unevenly distributed. The regularly staggered laminate as we discussed in the above section is the limit of Δ*s* = 0. If the value of the offset *s* has a distribution, the interface failure processes between adjacent platelets could be not simultaneous any more. The mechanical equilibrium configuration of the structure under uniaxial stretch is hard to be analytically solved but can be obtained by minimizing the total energy of the structure *E*^*tot*^ including the elastic energy in the all layers and the interface energies between all adjacent layers. The strong elastic interactions between adjacent layers can be taken into account by modeling the interlayer as a cohesive interface at which the interface energy is a functional of the displacement jump across the interface. The total energy can thus be written as a functional of the displacement in each layer *u*^(*i*)^ (See the details in the Supplementary Material Appendix B). Based on a gradient flow directed relaxation model, we assume that the energy minimization process is governed by the Ginzburg-Landau kinetic equation 
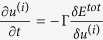
, where Γis a constant^34^. After the randomly staggered structure is generated (see the Figure S2 in the Supplementary Material), the evolution of the kinetic equation could track the stress field and the failure process in the laminate.

**Figure 6:**
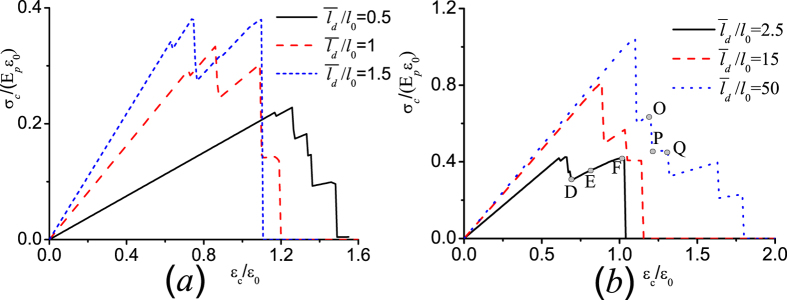
The calculated stress-strain curves demonstrating a so called “graceful failure” process in the ten-layers randomly staggered discontinuous laminate with a distributed offset *s* = [0.1, 0.06, 0.4, 0.2, 0.45, 0.4, 0.25, 0.2, 0.4] under different values of 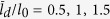 for (**a**), and 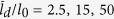 for (**b**).

**Figure 7:**
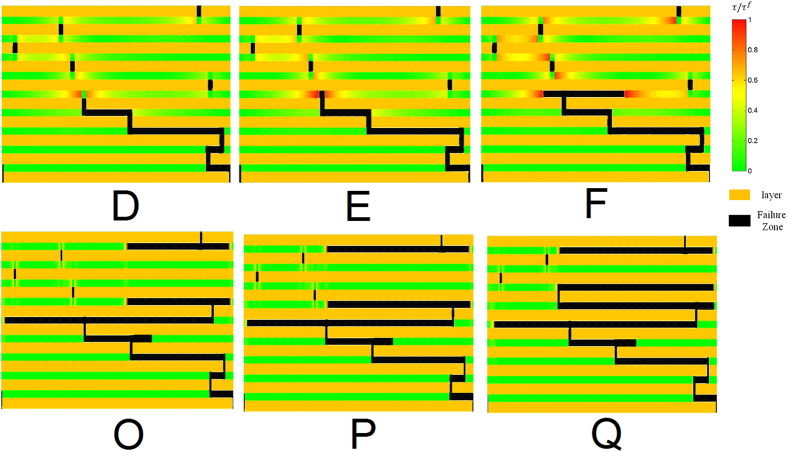
The snapshots of the laminated structure with propagation of the interface failure at the points D–F and O–P marked in Fig. 6(b).

**Figure 8:**
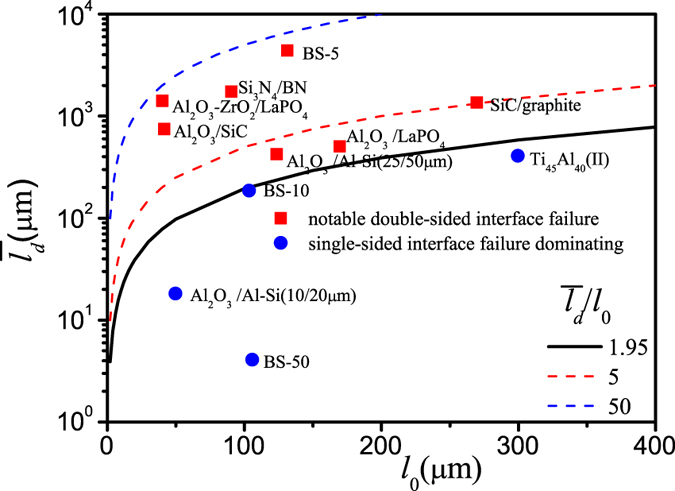
Two characteristic lengths coordinate the transition map from single-sided to double-sided interface failure wherein the input data is from the Table 1 in the Supplementary Material.

As we have checked (see the Figure S3 in the Supplementary Material), in the case of regularly staggered laminates with the offset *s* ≠ 0.5, i.e. *s* = 0.3, the overlapping length can be 0.3*l*_*p*_ or 0.7*l*_*p*_, the maximum interfacial stress appears at the interface zone with smaller overlapping length. Therefore the interface failure in regularly staggered laminates with the offset *s* ≠ 0.5, i.e. *s* = 0.49, prefers to occur only on the interface zone with smaller overlapping length instead of double-sided failure, and the enhancement of the resultant failure strain due to the single-sided interface failure drops to one-half, compared to the case of double-sided failure in all the interfaces (see the Figure S4 in the Supplementary Material). Figure 6 plot the calculated stress-strain curves of the ten-layers discontinuous laminated structure with a distributed offset in these layers *s* = [0.1, 0.06, 0.4, 0.2, 0.45, 0.4, 0.25, 0.2, 0.4] under different values of 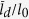. All the curves demonstrate a step-like or saw-tooth load-displacement response replicating the so called “graceful failure” widely observed in many tough laminates^1^,^15^,^16^,^17^,^18^,^19^. Figure 6(a) shows that the laminate with smaller average overlapping length has higher failure strain, in consistent with the previous study for the nacreous laminates using finite element method^48^ wherein the aspect ratio of the platelets is not big such that the shear stress is uniform with high stress-transfer efficiency^26^. Figure 6(b) indicates that the laminate with larger average overlapping length has higher failure strain, in good agreement with the previous experiment for the discontinuous laminate with ultrahigh aspect ratio of the platelet^35^. The results in Fig. 6 also demonstrate that the interface failure in the staggered laminate with a distributed offset tends to be a progressive layer-by-layer failure process. However, the overall mechanical response in the laminate cannot be simply viewed as the result of the superposition of propagation of interface failure involving different overlapping lengths (in which the average stress is 
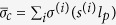
, and the average strain is the applied stretch *ε*_*c*_) since there are strong elastic interactions between adjacent layers. Figure 7 further shows the snapshots of the laminated structure during the interface failure process at the points D–F and O–P marked in Fig. 6(b). Figure 7D–F demonstrate that the strain hardening feature of the stress-strain curve in Fig. 6(b) is due to the fact that the adjacent interface starts to carry much more load with the increase of the applied stretch until it also fails completely. Figure 7O–Q elucidate that a sharp stress drop and the appearance of the platform in the stress-strain curve in Fig. 6(b) is due to the unstable and progressively stable propagation of the interface failure, respectively. In addition, we note that the single-sided interface failure dominating in the regularly staggered laminates with *s* ≠ 0.5 is mitigated in the randomly staggered laminates especially at the large value of 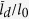. The results in Fig. 7 clearly show that with the increase of 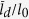 formation of double-sided interface failure is more favorable and it is not sensitive to the staggering arrangement any more. To check whether large value of 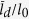 favors the double-sided interface failure or not, we estimate the points (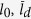) in many typical discontinuous laminates. The input data is from the Table 1 in the Supplementary Material Appendix D. The result in Fig. 8 indicates that with the increase of 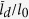 the single-sided interface failure mode exactly tends to change into the double-sided interface failure mode.

**Figure 9:**
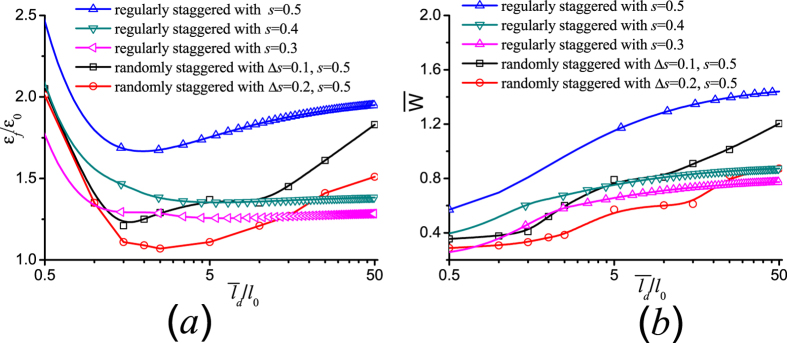
The calculated (**a**) failure strain and (**b**) effective toughness as a function of the average overlapping length in randomly staggered laminates compared with those of regularly staggered ones.

Obviously the double-sided interface failure could provide more tensile strain capacity and work of failure in comparison with the case of single-sided interface failure. Figure 9 plots the calculated failure strain and the work of failure as a function of 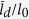 in randomly staggered laminates compared with those of regularly staggered ones. The result in Fig. 9 demonstrates that the failure strain and the dimensionless work of failure 
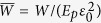
 with 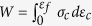 in the randomly staggered laminate can be larger than those in regularly staggered laminates with the offset *s* ≠ 0.5 wherein single-sided interface failure is dominant. The correlation between the large value of 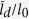 and synergistically increased failure strain, strength, and work of failure can now be understood based on the mechanism of the size-dependent interface failure.

## Discussion

In the above modeling and simulation, the interface is assumed to be brittle. For the interface involving significant plastic yielding^38^, we could model the interface to be elastic perfectly plastic^34^. Our calculated results indicate that interface plasticity promotes to homogenize the interfacial shear stress and the localization of the interfacial shear stress only appears in the nacreous composites with larger overlapping length. Interestingly the two size-dependent interface failure modes are still observed. It is understandable since the transition of the interface failure modes mainly depends on when the interfacial shear stress between laminae is uniform or highly localized. As we expect, the transition point *l*_*d*_/*l*_0_ = 1.95 shown in Fig. 4 shifts to the position with larger value of *l*_*d*_/*l*_0_ in the presence of notable interface plasticity as we have checked. In addition, we should note that the above conclusions are limited to the case without platelet fracture. They are valid only if the maximum tensile stress in the platelets is smaller than the fracture strength. The maximum tensile stress in the platelets for the case of regularly staggered laminates with *s* = 0.5 is 
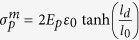
. Based on the Griffith theory, the fracture strength 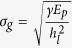 is saturated to the theoretic strength *σ*_th_ if the platelet’s thickness is below than a critical value 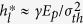 with *γ* the surface energy of the platelet^26^. Therefore, only if the parameters in the discontinuous laminate guarantee the condition 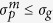 the platelet fracture will not occur. In addition, the real interface between adjacent platelet usually involves plasticity, friction and interlock, these factors lead to significant stress hardening and thus enhanced probability of the platelet fracture, as is observed in the nacre-mimetics composed of platelets with ultrahigh aspect ratios^33^.

In summary, using nonlinear shear-lag analysis on the discontinuous laminate we have shown that there are two kinds of propagation modes of the interface failure: unstable or progressively stable interface failure, dependent on whether the shear stress at the interface is uniform or localized. The transition from the uniform shear stress to the localized shear stress is determined by the critical ratio of the overlapping length to the characteristic length of the localized shear zone. If the value of the ratio is larger than two, the shear stress tends to be localized, and the interface failure tends to be progressively stable. These results have explained the non-monotonic size-dependent mechanical properties reported in various laminate composites. We expect that the size effect of the interface failure may provide guideline for rational design of strong and tough bioinspired laminates.

## Additional Information

**How to cite this article**: Song, Z.Q. *et al.* Interface failure modes explain non-monotonic size-dependent mechanical properties in bioinspired nanolaminates. *Sci. Rep.* **6**, 23724; doi: 10.1038/srep23724 (2016).

## Supplementary Material

Supplementary Information

## References

[b1] Clegg W. J. *et al.* A simple way to make tough ceramics. Nature 347, 455–457 (1990).

[b2] Chan H. M. Layered ceramics: processing and mechanical behavior. Annu. Rev. Mater. Sci. 27, 249–282 (1997).

[b3] Kim J. K. & Mai Y. W. Engineered interfaces in fiber reinforced composites. (Elsevier, 1998).

[b4] Meyers M. A., Chen P. Y., Lin A. Y. & Seki Y. Biological materials: structure and mechanical properties. Prog. Mater. Sci. 53, 1–206 (2008). 10.1016/j.jmbbm.2008.02.003 19627786

[b5] Cook J., Gordon J. E., Evans C. C. & Marsh D. M. A mechanism for the control of crack propagation in all-brittle systems. Proc. R. Soc. London, Ser. A 282, 508–520 (1964).

[b6] Ming-Yuan H. & Hutchinson J. W. Crack deflection at an interface between dissimilar elastic materials. Int. J. Solids Struct. 25, 1053–1067 (1989).

[b7] Hutchinson J. W. & Suo Z. Mixed mode cracking in layered materials. Adv. Appl. Mech. 29, 191 (1992).

[b8] Ritchie R. O. The conflicts between strength and toughness. Nat. Mater. 10, 817–822 (2011). 22020005 10.1038/nmat3115

[b9] Wang R. & Gupta H. S. Deformation and fracture mechanisms of bone and nacre. Annu. Rev. Mater. Res. 41, 41–73 (2011).

[b10] Barthelat F. & Rabiei R. Toughness amplification in natural composites. J. Mech. Phys. Solids 59, 829–840 (2011).

[b11] Shao Y., Zhao H. P., Feng X. Q. & Gao H. Discontinuous crack-bridging model for fracture toughness analysis of nacre. J. Mech. Phys. Solids 60, 1400–1419 (2012).

[b12] Chen Z. & Mecholsky J. J. Control of strength and toughness of ceramic/metal laminates using interface design. J. Mater. Res. 8, 2362–2369 (1993).

[b13] Hwu K. L. & Derby B. Fracture of metal/ceramic laminates—I. transition from single to multiple cracking. Acta Mater. 47, 529–543 (1999).

[b14] Yamaguchi M., Inui H. & Ito K. High-temperature structural intermetallics. Acta Mater. 48, 307–322 (2000).

[b15] Peng L. M., Li H. & Wang J. H. Processing and mechanical behavior of laminated titanium–titanium tri-aluminide (Ti–Al_3_Ti) composites. Mat. Sci. Eng. A 406, 309–318 (2005).

[b16] Kovar D., Thouless M. D. & Halloran J. W. Crack deflection and propagation in layered silicon nitride/boron nitride ceramics. J. Am. Ceram. Soc. 81, 1004–1112 (1998).

[b17] She J., Inoue T. & Ueno K. Multilayer Al_2_O_3_/SiC ceramics with improved mechanical behavior. J. Eur. Ceram. Soc. 20, 1771–1775 (2000).

[b18] Li X. *et al.* Micro/nanoscale mechanical characterization and in situ observation of cracking of laminated Si_3_N_4_/BN composites. Mat. Sci. Eng. C 28, 1501–1508 (2008).

[b19] Wang C. A. *et al.* Biomimetic structure design—a possible approach to change the brittleness of ceramics in nature. Mat. Sci. Eng. C 11, 9–12 (2000).

[b20] Currey J. D. Mechanical properties of mother of pearl in tension. Proc. R. Soc. Lond. B 196, 443–463 (1977).

[b21] Espinosa H. D., Rim J. E., Barthelat F. & Buehler M. J. Merger of structure and material in nacre and bone–Perspectives on de novo biomimetic materials. Prog. Mater. Sci. 54, 1059–1100 (2009).

[b22] Wang J., Cheng Q. & Tang Z. Layered nanocomposites inspired by the structure and mechanical properties of nacre. Chem. Soc. Rev. 41, 1111–1129 (2012). 21959863 10.1039/c1cs15106a

[b23] Yao H. B. *et al.* 25th anniversary article: artificial carbonate nanocrystals and layered structural nanocomposites inspired by nacre: synthesis, fabrication and applications. Adv. Mater. 26, 163–188 (2014). 24338814 10.1002/adma.201303470

[b24] Bouville F. *et al.* Strong, tough and stiff bioinspired ceramics from brittle constituents. Nat. Mater. 13, 508–514 (2014). 24658117 10.1038/nmat3915

[b25] Nassif N. *et al.* Amorphous layer around aragonite platelets in nacre. Proc. Natl. Acad. Sci. USA 102, 12653–12655 (2005). 16129830 10.1073/pnas.0502577102 PMC1200266

[b26] Gao H. *et al.* Materials become insensitive to flaws at nanoscale: lessons from nature. Proc. Natl. Acad. Sci. USA 100, 5597–5600 (2003). 12732735 10.1073/pnas.0631609100 PMC156246

[b27] Chen B., Wu P. D. & Gao H. A characteristic length for stress transfer in the nanostructure of biological composites. Compos. Sci. Technol. 69, 1160–1164 (2009).

[b28] Zhang Z. Q. *et al.* Mechanical properties of unidirectional nanocomposites with non-uniformly or randomly staggered platelet distribution. J. Mech. Phys. Solids 58, 1646–1660 (2010).

[b29] Wei X., Naraghi M. & Espinosa H. D. Optimal length scales emerging from shear load transfer in natural materials: application to carbon-based nanocomposite design. ACS Nano 6, 2333–2344 (2012). 22316210 10.1021/nn204506d

[b30] Sakhavand N., Muthuramalingam P. & Shahsavari R. Toughness governs the rupture of the interfacial H-bond assemblies at a critical length scale in hybrid materials. Langmuir 29, 8154–8163 (2013). 23713817 10.1021/la4014015

[b31] Liu Y. & Xu Z. Multimodal and self-healable interfaces enable strong and tough graphene-derived materials. J. Mech. Phys. Solids 70, 30–41 (2014).

[b32] Pimenta S. & Robinson P. An analytical shear-lag model for composites with ‘brick-and-mortar’ architecture considering non-linear matrix response and failure. Compos. Sci. Technol. 104, 111–124 (2014).

[b33] Das P. *et al.* Nacre-mimetics with synthetic nanoclays up to ultrahigh aspect ratios. Nat. Commun. 6, 5967 (2015). 25601360 10.1038/ncomms6967

[b34] Ni Y. *et al.* Optimization design of strong and tough nacreous nanocomposites through tuning characteristic lengths. J. Mech. Phys. Solids 81, 41–57 (2015).

[b35] Czél G., Pimenta S., Wisnom M. R. & Robinson P. Demonstration of pseudo-ductility in unidirectional discontinuous carbon fibre/epoxy prepreg composites. Compos. Sci. Technol. 106, 110–119 (2015).

[b36] Sakhavand N. & Shahsavari R. Universal composition–structure–property maps for natural and biomimetic platelet–matrix composites and stacked heterostructures. Nat. Commun. 6, 6523 (2015). 25774944 10.1038/ncomms7523

[b37] Zuo S. & Wei Y. Effective elastic modulus of bone-like hierarchical materials. Acta Mechanica Solida Sinica 20, 198–205 (2007).

[b38] Chen B., Wu P. D. & Gao H. A characteristic length for stress transfer in the nanostructure of biological composites. Compos. Sci. Technol. 69, 1160–1164 (2009).

[b39] Liu G. *et al.* Analytical solutions of the displacement and stress fields of the nanocomposite structure of biological materials. Compos. Sci. Technol. 71, 1190–1195 (2011).

[b40] Bar-On B. & Wagner H. D. Mechanical model for staggered bio-structure. J. Mech. Phys. Solids 59, 1685–1701 (2011).

[b41] Goland M. & Reissner E. The stresses in cemented joints. J. Appl Mech. 66, A17–A27 (1944).

[b42] Cox H. L. The elasticity and strength of paper and other fibrous materials. Br. J. Appl. Phys. 3, 72 (1952).

[b43] Nairn J. A. On the use of shear-lag methods for analysis of stress transfer in unidirectional composites. Mech. Mater. 26, 63–80 (1997).

[b44] Begley M. R. *et al.* Micromechanical models to guide the development of synthetic ‘brick and mortar’ composites. J. Mech. Phys. Solids 60, 1545–1560 (2012).

[b45] Parmigiani J. P. & Thouless M. D. The roles of toughness and cohesive strength on crack deflection at interfaces. J. Mech. Phys. Solids 54, 266–287 (2006).

[b46] Pavia F. & Curtin W. A. Molecular modeling of cracks at interfaces in nanoceramic composites. J. Mech. Phys. Solids 61, 1971–1982 (2013).

[b47] Tomaszewski H. *et al.* Multilayer ceramic composites with high failure resistance. J. Eur. Ceram. Soc. 27, 1373–1377(2007).

[b48] Askarinejad S. & Rahbar N. Toughening mechanisms in bioinspired multilayered materials. J. R. Soc. Interface 12, 20140855 (2014). 25551150 10.1098/rsif.2014.0855 PMC4277076

