# Supplementary material for: Interface failure modes explain non-monotonic size-dependent mechanical properties in bioinspired nanolaminates
Source: Sci Rep. 2016 Mar 31;6:23724. doi: 10.1038/srep23724 (PMC4814825; doi:10.1038/srep23724)
Supplement: Supplementary Information [file srep23724-s1.doc]

Supplementary Materials for “Interface failure modes explain non-monotonic size-dependent mechanical properties in bioinspired nanolaminates” by Z.Q. Song, Y. Ni[[1]](#footnote-2)*, L.M. Peng, H.Y. Liang, L.H.He.

**Contents:**

1. **Appendix A: A nonlinear shear lag model for regularly discontinuous laminated structure without offset**
2. **Appendix B: Numerical Simulation for discontinuous laminated structure**
3. **Appendix C: The localization of shear stress and the mode of interface failure**
4. **Appendix D: Data of laminar materials**

**1. Appendix A: A nonlinear shear lag model for regularly discontinuous laminated structure without offset**

Figure S1 the sketch of RVE for the interface failure process, under unidirectional stretch, and the interface zone divided into failure zone, localized shear zone and zero shear stress zone, according to the interfacial shear conditions.

Figure S1 shows a representative volume element (RVE) of regularly discontinuous laminated structure, whose interface is divided into three parts, including failure zone, localized shear zone, and zero shear stress zone. The discontinuous laminated structure has some characters: the thickness of interlayer $h_{\mathrm{int}}$ is much smaller than the thickness of the platelets $h_{l}$, the length of the platelets $l_{p}$ is much larger than the thickness of the platelets $h_{l}$. The shear strength of interlayer $\tau^{f}$ is much smaller than the failure strength of the platelets $\sigma_{p}^{f}$, and interlayer easily undergoes shear-like deformation. The platelets are brittle and they only have elastic deformation with the elastic modulus $E_{p}$. The mechanical response of such structures is obtained by performing nonlinear shear-lag analysis for RVE. An elastic interface is used to characterize the lateral interaction between bricks via the shear deformation of the interlayer. It can reproduce experimentally observed parameters like elastic modulus, post-yield slope, failure point and shear strength by fitting. Here as shown in Figure 1 the interface is assumed to be elastic, with shear modulus $G_{\mathrm{int}}$, shear strength $\tau^{f}$ and failure strain $\gamma_{e}^{f}$, for simplicity. The overlap length, which is also the distance of adjacent crack, of the platelets, is satisfied with $l_{d}=0.5l_{p}$ in regularly discontinuous laminated structure without offset.

The governing equations for the elastic zone (including shear stress zone and zero shear stress zone) of RVE of regularly discontinuous laminated structure are

$$\left\{\begin{matrix} \tau=G_{\mathrm{int}}\gamma\text{ } \\ \frac{\partial\sigma}{\partial x}+\frac{\partial\tau}{\partial y}=0\text{ } \end{matrix}\right.\text{ }a<x<0.5l_{p}-a.$$

The first expression describes the interlayer, and the second one describes platelet #1. Substituting $\frac{\partial\tau}{\partial y}=\frac{2\tau}{h_{l}}$, $\sigma=E_{p}\frac{\partial u_{1\text{a}}}{\partial x}$ for platelet #1, and $\frac{\partial\tau}{\partial y}=-\frac{2\tau}{h_{l}}$, $\sigma=E_{p}\frac{\partial u_{2}}{\partial x}$ for platelet #2 into Eq. we have

$$\left\{\begin{matrix} G_{\mathrm{int}}\frac{u_{2}\left(x\right)-u_{1}\left(x\right)}{h_{\mathrm{int}}}+\frac{1}{2}h_{l}E_{p}u''_{1}\left(x\right)=0\text{ } \\ G_{\mathrm{int}}\frac{u_{2}\left(x\right)-u_{1}\left(x\right)}{h_{\mathrm{int}}}-\frac{1}{2}h_{l}E_{p}u''_{2}\left(x\right)=0\text{ } \end{matrix}\right.a<x<0.5l_{p}-a.$$

The failure zone of platelet #1and platelet #2 is expressed as

$$\left\{\begin{matrix} \frac{1}{2}h_{l}E_{p}u''_{1}\left(x\right)=0 \\ \frac{1}{2}h_{l}E_{p}u''_{2}\left(x\right)=0 \end{matrix}\right.\text{ }0\leq x\leq a\text{ and 0.5}l_{p}-a\leq x\leq0.5l_{p}\text{. }$$

We set $\bar{a}=\frac{a}{l_{p}}$ and $\bar{x}=\frac{x}{l_{p}}$. From Eq. and Eq. , the normalized equations are given as

$$\left\{\begin{matrix} \bar{u}''_{1}=0,\text{ }0\leq\bar{x}\leq\bar{a}\text{ or 0.5}-\bar{a}\leq\bar{x}\leq0.5 \\ \bar{u}''_{1}=2k_{2}^{2}\left(\bar{u}_{1}-\bar{u}_{2}\right),\text{ }\bar{a}\leq\bar{x}\leq0.5-\bar{a} \\ \bar{u}''_{2}=0,\text{ }0\leq\bar{x}\leq\bar{a}\text{ or 0.5}-\bar{a}\leq\bar{x}\leq0.5 \\ \bar{u}''_{2}=2k_{2}^{2}\left(\bar{u}_{2}-\bar{u}_{1}\right),\text{ }\bar{a}\leq\bar{x}\leq0.5-\bar{a} \end{matrix}\right.\text{ }$$

where $\alpha=\sqrt{\frac{\tau^{f}l_{p}}{E_{p}h_{l}}}$ and $k_{2}=\sqrt{\frac{G_{\mathrm{int}}l_{p}^{2}}{E_{p}h_{l}h_{\mathrm{int}}}}=\frac{2l_{d}}{l_{0}}$. $l_{0}=\sqrt{\frac{E_{p}h_{l}h_{\mathrm{int}}}{G_{\mathrm{int}}}}$ is the critical length.

The solutions of Eq. can be written as

$$\left\{\begin{matrix} \bar{u}_{1}=c_{1}\bar{x}+c_{2},\text{ }0\leq\bar{x}\leq\bar{a} \\ \bar{u}_{1}=c_{3}+c_{4}\bar{x}-c_{5}\text{sinh}\left(2k_{2}\bar{x}\right)-c_{6}\text{cosh}\left(2k_{2}\bar{x}\right),\text{ }\bar{a}\leq\bar{x}\leq0.5-\bar{a} \\ \bar{u}_{1}=c_{7}\bar{x}+c_{8},\text{ 0.5}-\bar{a}\leq\bar{x}\leq0.5 \\ \bar{u}_{2}=c_{9}\bar{x}+c_{10},\text{ }0\leq\bar{x}\leq\bar{a} \\ \bar{u}_{2}=c_{3}+c_{4}\bar{x}+c_{5}\text{sinh}\left(2k_{2}\bar{x}\right)+c_{6}\text{cosh}\left(2k_{2}\bar{x}\right),\text{ }\bar{a}\leq\bar{x}\leq0.5-\bar{a} \\ \bar{u}_{2}=c_{11}\bar{x}+c_{12},\text{ 0.5}-\bar{a}\leq\bar{x}\leq0.5 \end{matrix}\right.\text{ }$$

The stress and displacement in the platelets #1 and #2 are continuous at $\bar{x}=\bar{a}$ and $\bar{x}=\left(0.5-\bar{a}\right)$. They lead to eight equations

$$\begin{matrix} \bar{u}'_{1}\left(\bar{x}=\bar{a}_{-}\right)=\bar{u}'_{1}\left(\bar{x}=\bar{a}_{+}\right) \\ \bar{u}_{1}\left(\bar{x}=\bar{a}_{-}\right)=\bar{u}_{1}\left(\bar{x}=\bar{a}_{+}\right) \\ \bar{u}'_{1}\left[\bar{x}=\left(0.5-\bar{a}\right)_{-}\right]=\bar{u}'_{1}\left[\bar{x}=\left(0.5-\bar{a}\right)_{+}\right] \\ \bar{u}_{1}\left[\bar{x}=\left(0.5-\bar{a}\right)_{-}\right]=\bar{u}_{1}\left[\bar{x}=\left(0.5-\bar{a}\right)_{+}\right] \\ \bar{u}'_{2}\left(\bar{x}=\bar{a}_{-}\right)=\bar{u}'_{2}\left(\bar{x}=\bar{a}_{+}\right) \\ \bar{u}_{2}\left(\bar{x}=\bar{a}_{-}\right)=\bar{u}_{2}\left(\bar{x}=\bar{a}_{+}\right) \\ \bar{u}'_{2}\left[\bar{x}=\left(0.5-\bar{a}\right)_{-}\right]=\bar{u}'_{2}\left[\bar{x}=\left(0.5-\bar{a}\right)_{+}\right] \\ \bar{u}_{2}\left[\bar{x}=\left(0.5-\bar{a}\right)_{-}\right]=\bar{u}_{2}\left[\bar{x}=\left(0.5-\bar{a}\right)_{+}\right] \end{matrix}$$

There are four boundary conditions for the RVE

$\bar{u}_{1}\left(0\right)=0$, $\bar{u}'_{2}\left(0\right)=0$,$\bar{u}'_{1}\left(\bar{a}\right)=\bar{u}'_{2}\left(0.5-\bar{a}\right)$,$\bar{u}_{2}\left(0.5\right)=\bar{\Delta}$

Substituting Eqs. - into Eq., $c_{1}$ to $c_{12}$ can be determined by $\bar{\Delta}$, $k_{2}$, and $\alpha$.

$$\begin{matrix} c_{1}=\frac{\bar{\Delta}k_{2}\mathrm{sech}^{2}\left[k_{2}\left(2\bar{a}-0.5\right)\right]\sinh\left[2k_{2}\left(2\bar{a}-0.5\right)\right]}{-1+k_{2}\left(2\bar{a}+0.5\right)\tanh\left[k_{2}\left(2\bar{a}-0.5\right)\right]} \\ c_{2}=0 \\ c_{3}=\frac{\bar{\Delta}\left\{\cosh\left[k_{2}\left(2\bar{a}-0.5\right)\right]-2\bar{a}k_{2}\sinh\left[k_{2}\left(2\bar{a}-0.5\right)\right]\right\}}{2\left\{\cosh\left[k_{2}\left(2\bar{a}-0.5\right)\right]-k_{2}\left(2\bar{a}+0.5\right)\sinh\left[k_{2}\left(2\bar{a}-0.5\right)\right]\right\}} \\ c_{4}=\frac{\bar{\Delta}k_{2}\mathrm{sech}^{2}\left[k_{2}\left(2\bar{a}-0.5\right)\right]\sinh\left[2k_{2}\left(2\bar{a}-0.5\right)\right]}{2\left\{-1+k_{2}\left(2\bar{a}+0.5\right)\tanh\left[k_{2}\left(2\bar{a}-0.5\right)\right]\right\}} \\ c_{5}=\frac{\bar{\Delta}\mathrm{sech}^{2}\left[k_{2}\left(2\bar{a}-0.5\right)\right]\left\{\sinh\left(2\bar{a}k_{2}\right)+\sinh\left[2k_{2}\left(0.5-\bar{a}\right)\right]\right\}}{4\left\{-1+k_{2}\left(2\bar{a}+0.5\right)\tanh\left[k_{2}\left(2\bar{a}-0.5\right)\right]\right\}} \\ c_{6}=-\frac{\bar{\Delta}\mathrm{sech}^{2}\left[k_{2}\left(2\bar{a}-0.5\right)\right]\left\{\cosh\left(2\bar{a}k_{2}\right)+\cosh\left[2k_{2}\left(0.5-\bar{a}\right)\right]\right\}}{4\left\{-1+k_{2}\left(2\bar{a}+0.5\right)\tanh\left[k_{2}\left(2\bar{a}-0.5\right)\right]\right\}} \\ c_{7}=0 \\ c_{8}=-\frac{\bar{\Delta}k_{2}\sinh\left[k_{2}\left(2\bar{a}-0.5\right)\right]\mathrm{csch}\left[2k_{2}\left(2\bar{a}-0.5\right)\right]}{\mathrm{csch}\left[k_{2}\left(2\bar{a}-0.5\right)\right]-k_{2}\left(2\bar{a}+0.5\right)\mathrm{sech}\left[k_{2}\left(2\bar{a}-0.5\right)\right]} \\ c_{9}=0 \\ c_{10}=\frac{\bar{\Delta}\left\{\cosh\left[k_{2}\left(2\bar{a}-0.5\right)\right]-2\bar{a}k_{2}\sinh\left[k_{2}\left(2\bar{a}-0.5\right)\right]\right\}}{\cosh\left[k_{2}\left(2\bar{a}-0.5\right)\right]-k_{2}\left(2\bar{a}+0.5\right)\sinh\left[k_{2}\left(2\bar{a}-0.5\right)\right]} \\ c_{11}=\frac{\bar{\Delta}k_{2}\mathrm{sech}^{2}\left[k_{2}\left(2\bar{a}-0.5\right)\right]\sinh\left[2k_{2}\left(2\bar{a}-0.5\right)\right]}{-1+k_{2}\left(2\bar{a}+0.5\right)\tanh\left[k_{2}\left(2\bar{a}-0.5\right)\right]} \\ c_{12}=\frac{\bar{\Delta}\left\{\cosh\left[k_{2}\left(2\bar{a}-0.5\right)\right]-k_{2}\left(2\bar{a}-0.5\right)\sinh\left[k_{2}\left(2\bar{a}-0.5\right)\right]\right\}}{\cosh\left[k_{2}\left(2\bar{a}-0.5\right)\right]-k_{2}\left(2\bar{a}+0.5\right)\sinh\left[k_{2}\left(2\bar{a}-0.5\right)\right]} \end{matrix}$$

The value of $\bar{a}$ can be determined by assumption of the shear stress $\bar{\tau}=\tau/\tau^{f}=-\frac{1}{2\alpha^{2}}\bar{u''}_{1}$at the point $\bar{x}=\bar{a}_{+}$. From Eq. , the shear stress of interface between platelet #1a and platelet #2 is

$$\bar{\tau}=\left\{\begin{matrix} 0,\text{ }0\leq\bar{x}\leq\bar{a} \\ \frac{2k_{2}^{2}}{\alpha^{2}}\left[c_{5}\text{sinh}\left(2k_{2}\bar{x}\right)+c_{6}\text{cosh}\left(2k_{2}\bar{x}\right)\right],\bar{a}\leq\bar{x}\leq0.5-\bar{a} \\ 0,\text{ 0.5}-\bar{a}\leq\bar{x}\leq0.5 \end{matrix}\right.$$

According to Eq. the shear stress at the point $\bar{x}=\bar{a}$ requires $\bar{\tau}\left(\bar{x}=\bar{a}_{+}\right)=1$so

$$\frac{2k_{2}^{2}}{\alpha^{2}}\left[c_{5}\text{sinh}\left(2k_{2}\bar{a}\right)+c_{6}\text{cosh}\left(2k_{2}\bar{a}\right)\right]=1$$

Substituting the forms of $c_{5}$ and $c_{6}$ in Eq. into Eq., we have

$$\bar{\Delta}=\frac{\varepsilon_{0}}{k_{2}}\left\{1-k_{2}\left(2\bar{a}+0.5\right)\tanh\left[k_{2}\left(2\bar{a}-0.5\right)\right]\right\}$$

where we define the critical strain $\varepsilon_{0}=\frac{\alpha^{2}}{k_{2}}=\tau^{f}\sqrt{\frac{h_{\mathrm{int}}}{E_{p}G_{\mathrm{int}}h_{l}}}$, which is only related to the mechanical properties of material components.

In the regular structure, the composite strain $\varepsilon_{c}$ is satisfied with $\varepsilon_{c}=2\bar{\Delta}$ so

$$\varepsilon_{c}=\frac{2\varepsilon_{0}}{k_{2}}\left\{1-k_{2}\left(2\bar{a}+0.5\right)\tanh\left[k_{2}\left(2\bar{a}-0.5\right)\right]\right\}$$

The applied stress is satisfied with $\sigma_{c}=2\int_{0}^{0.5} \sigma d\bar{x}$, so it is expressed as

$$\sigma_{c}=-E_{p}\varepsilon_{0}\tanh\left[k_{2}\left(2\bar{a}-0.5\right)\right]$$

When failure occurs on the interface, the composite strain corresponds to yield strain

$$\varepsilon_{y}=\frac{2\varepsilon_{0}}{k_{2}}\left[1+0.5k_{2}\tanh\left(0.5k_{2}\right)\right]$$

The failure strain of composite is defined as the max strain obtained in the process of interface failure. In order to obtain the failure strain $\varepsilon_{f}$, we should calculate the peak value in Eq.. The differential $\frac{d\varepsilon_{c}}{d\bar{a}}$ can expressed as

$$\frac{d\varepsilon_{c}}{d\bar{a}}=-4\varepsilon_{0}\left\{k_{2}\left(2\bar{a}+0.5\right)\mathrm{sech}^{2}\left[k_{2}\left(2\bar{a}-0.5\right)\right]+\tanh\left[k_{2}\left(2\bar{a}-0.5\right)\right]\right\}$$

when $\frac{d\varepsilon_{c}}{d\bar{a}}>0$, applied strain increase, the failure zone increases stably. The critical condition is $\left.\frac{d\varepsilon_{c}}{d\bar{a}}\right|_{\bar{a}=\bar{a}_{0}}=0$, so we obtain

$$2k_{2}\left(2\bar{a}_{0}+0.5\right)+\sinh\left[2k_{2}\left(2\bar{a}_{0}-0.5\right)\right]=0$$

From Eq., we can get the critical value $\bar{a}_{0}$, we put the value $\bar{a}_{0}$ into Eq., we can get the failure strain $\varepsilon_{f}$, and it is expressed as

$$\varepsilon_{f}=\frac{2\varepsilon_{0}}{k_{2}}\left\{1-k_{2}\left(2\bar{a}_{0}+0.5\right)\tanh\left[k_{2}\left(2\bar{a}_{0}-0.5\right)\right]\right\}$$

The max stress at point $\bar{x}=0$in brick #1a is the max stress, so the max stress is

$$\sigma_{p}^{m}=-2E_{p}\varepsilon_{0}\tanh\left[k_{2}\left(2\bar{a}-0.5\right)\right]$$

so when $k_{2}\to\infty$, the max stress of brick is $\sigma_{p}^{m}=2E_{p}\varepsilon_{0}$.

According to the applied stress and applied strain, we define the effective toughness $W=\int_{0}^{\varepsilon_{f}} \sigma_{c}d\varepsilon_{c}$, which represents the total energy absorbed before laminated structure thoroughly failed; and the uniformed effective toughness is shown as $\bar{W}=W/\left(E_{p}\varepsilon_{0}^{2}\right)$.

**2. Appendix B: Numerical Simulation for discontinuous laminated structure**

We use representative volume element (RVE) with periodic boundary conditions to simulate the randomly discontinuous laminated structure. The RVE we considered here has the size of $L_{x}\times L_{y}$, with $L_{x}=l_{p}$ and $L_{y}=50h_{l}$. Since each platelet has the thickness of $h_{l}$, there are 50 layers, which number guarantees the staggering randomness. After the randomly discontinuous laminated structure is generated, the mechanical equilibrium configuration of the laminated structure under uniaxial stretch can be obtained by minimizing the total energy of the discontinuous laminated structure including the elastic energy in the all platelet layers $\sum_{i=1}^{50} E_{el}^{\left(i\right)}$and the interface energy between every two platelet layers $\sum_{i=1}^{50} E_{\mathrm{int}}^{\left(i\right)}$.

$$E^{tot}=\sum_{i=1}^{50} \left(E_{el}^{\left(i\right)}+E_{\mathrm{int}}^{\left(i\right)}\right)$$

The elastic energy of the platelet layer $i$ has the form:

$$E_{el}^{\left(i\right)}=\frac{1}{2}\int c\left(x\right)^{\left(i\right)}\left[\varepsilon\left(x\right)^{\left(i\right)}\right]^{2}h_{l}dx$$

where $c\left(x\right)^{\left(i\right)}=E_{p}$ when $x$ is in the segment otherwise $c\left(x\right)^{\left(i\right)}=0$, and $\varepsilon\left(x\right)^{\left(i\right)}=\partial u^{\left(i\right)}/\partial x$. Using a cohesive zone model, the interface energy adjacent to the layer $i$ can be written as

$$E_{\mathrm{int}}^{\left(i\right)}=\frac{h_{\mathrm{int}}}{2}\left[\iint\tau^{\left(i+1\right)}d\gamma^{\left(i+1\right)}dx+\iint\tau^{\left(i\right)}d\gamma^{\left(i\right)}dx\right]$$

where the shear strain $\gamma^{\left(i\right)}=\frac{u^{\left(i\right)}-u^{\left(i-1\right)}}{h_{\mathrm{int}}}$ and

$$\tau^{\left(i\right)}=\left\{\begin{matrix} \frac{G_{\mathrm{int}}^{\left(i\right)}}{h_{\mathrm{int}}}\left[u^{\left(i\right)}-u^{\left(i-1\right)}\right],\text{ }\left|\gamma^{\left(i\right)}\right|<\gamma_{e}^{c} \\ \left(\tau^{f}\right)^{\left(i\right)}sign\left(\gamma^{\left(i\right)}\right),\text{ }\gamma_{e}^{c}<\left|\gamma^{\left(i\right)}\right|<\gamma_{p}^{f} \\ 0,\text{ }\left|\gamma^{\left(i\right)}\right|\geq\gamma_{p}^{f} \end{matrix}\right.$$

Based on a gradient flow directed relaxation model, we assumed that the energy minimization process is governed by the Ginzburg-Landau kinetic equation,

$$\frac{\partial u^{\left(i\right)}}{\partial t}=-\Gamma\frac{\delta E^{tot}}{\delta u^{\left(i\right)}}$$

The equilibrium configuration is obtained after there is a steady solution of Eq., which reduces to Eqs. and .

Substituting Eqs. - into Eq. with $\bar{c}^{\left(i\right)}=\frac{c^{\left(i\right)}}{E_{p}}$, $\bar{x}=\frac{x}{l_{p}}$, $\bar{u}^{\left(i\right)}\left(\bar{x}\right)=\frac{u^{\left(i\right)}\left(x\right)}{l_{p}}$, $\bar{L}_{x}=L_{x}/l_{p}=1$, and the normalized kinetic equation is rewritten as：

$$\frac{\partial\bar{u}^{\left(i\right)}}{\partial t}=-\bar{\Gamma}\left\{-\frac{\partial}{\partial\bar{x}}\left(\bar{c}^{\left(i\right)}\frac{\partial\bar{u}^{\left(i\right)}}{\partial\bar{x}}\right)+\left[\bar{\tau}^{\left(i\right)}-\bar{\tau}^{\left(i+1\right)}\right]\right\}$$

where $\bar{\Gamma}=\frac{\Gamma E_{p}h_{l}}{l_{p}^{2}}$is assumed to be 1. A finite difference method is adopted to solve Eq. for each layer given the boundary condition

$$\left\{\begin{matrix} \frac{\partial\bar{u}^{\left(i\right)}\left(0\right)}{\partial\bar{x}}=\frac{\partial\bar{u}^{\left(i\right)}\left(\bar{L}_{x}\right)}{\partial\bar{x}}\text{, }\bar{u}^{\left(i\right)}\left(\bar{L}_{x}\right)-\bar{u}^{\left(i\right)}\left(0\right)=\bar{\Delta}_{0},\text{ if }\bar{c}^{\left(i\right)}\left(0\right)=1 \\ \frac{\partial\bar{u}^{\left(i\right)}\left(0\right)}{\partial\bar{x}}=\frac{\partial\bar{u}^{\left(i\right)}\left(\bar{L}_{x}\right)}{\partial\bar{x}}=0,\text{ }\begin{matrix} \begin{matrix} \begin{matrix} & \end{matrix} & \end{matrix} & & \end{matrix}\text{ if }\bar{c}^{\left(i\right)}\left(0\right)=0 \end{matrix}\right.$$

with the initial condition

$$\bar{u}^{\left(i\right)}\left(\bar{x}\right)=\frac{\bar{x}}{\bar{L}_{x}}\bar{\Delta}_{0}$$

where $\bar{\Delta}_{0}=\Delta_{0}/l_{p}$.

In the randomly discontinuous laminated structure, we generate the randomness by setting a standard deviation $\Delta s$, and in this case, the average overlap length $\bar{l}_{d}$ is satisfied with $\bar{l}_{d}=\bar{s}l_{p}$ where $\bar{s}=0.5$ is the mean overlap factor. Figure S2 shows typical generated randomly staggered structures with (a)$\Delta s=0.04$, (b) $\Delta s=0.16$ during our numerical simulations.


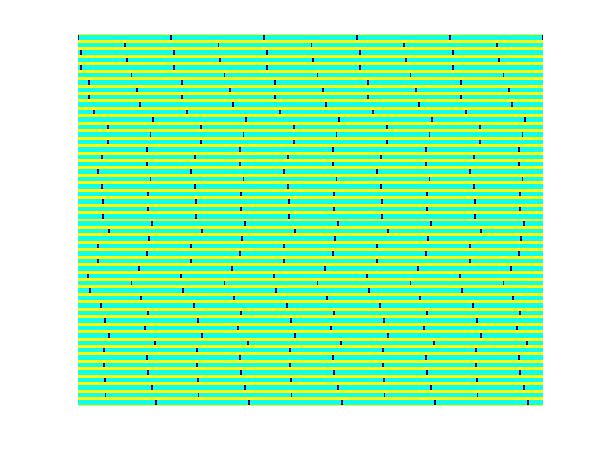

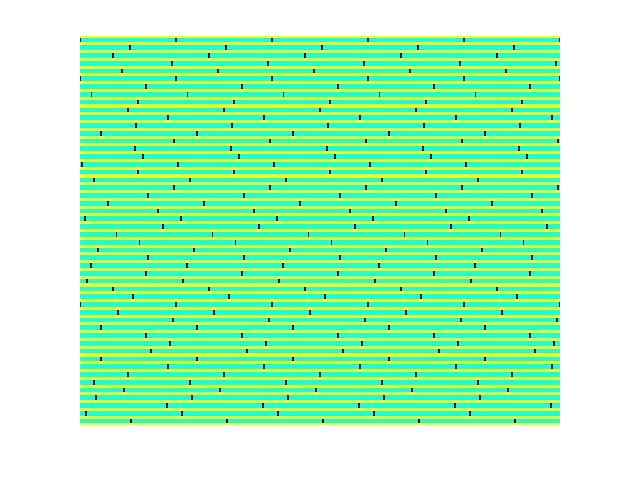


(a) (b)

Figure S2 Generated randomly staggered structure by a normal distribution with the mean offset $\bar{s}=0.5$ and a standard deviation $\Delta s$ for (a)$\Delta s=0.04$, for (b) $\Delta s=0.16$.

At first, we simulate the regularly discontinuous laminated structure with offset in which the overlap length $l_{d}$ is satisfied with $l_{d}=0.3l_{p}$ (the overlap factor is $s=0.3$). Figure S3 plots the absolute shear stress distribution along interface with different overlap lengths in the condition that peak shear stress reaches the shear strength. Notably comparative shear stress indicates that peak shear stress always occurs on the short side, where the interface failure arises after shear stress exceeds the shear strength.

Figure S3 the typical absolute shear stress distribution along interface in regularly discontinuous laminated structure with offset ($s=0.3$).

**3. Appendix C: The localization of shear stress and the mode of interface failure**

When the failure zone begins to progress, the shear stress in non-failure zone along interface is expressed as

$$\frac{\tau\left(\bar{x}\right)}{\tau^{f}}=\frac{-\sinh\left(k_{2}\right)\sinh\left(2k_{2}\bar{x}\right)+\left[1+\cosh\left(k_{2}\right)\right]\cosh\left(2k_{2}\bar{x}\right)}{1+\cosh\left(k_{2}\right)}$$

The minimum shear stress occurs in the middle of the RVE, where $\bar{x}=0.25$, so it is expressed as

$$\frac{\tau\left(0.25\right)}{\tau^{f}}=\frac{1}{\cosh\left(0.5k_{2}\right)}$$

where $\cosh\left(0.5k_{2}\right)=\cosh\left(l_{d}/l_{0}\right)$. The shear stress in the middle of RVE decays exponentially with the increase of overlap length $l_{d}$, and attenuation length of minimum shear stress is $l_{0}$ as the independent variable $l_{d}$. Similar result is also uncovered in figure S2.

Figure S4 shows the stress-strain curve and microstructure of regularly discontinuous laminated structure without offset ($s=0.5$) and regular discontinuous laminated structure with offset ($s=0.49$). The single-sided interface failure is occurred on the short overlap sides, for these short sides initially reach the shear strength, in regularly discontinuous laminated structure with offset, which, as a result, has a smaller failure strain. While in randomly discontinuous laminated structure the shear stress gradually increases until it reaches the shear strength with applied strain increasing, but it will redistribute when nearby interface totally fails, shown in figure S5. During the redistributed process, the peak shear stress of long overlap side may exceed that of the short one and, even in some cases, the peak shear stress of long overlap side outgrows the shear strength which lead to the interface failure, shown in figure S5(d).

(a)


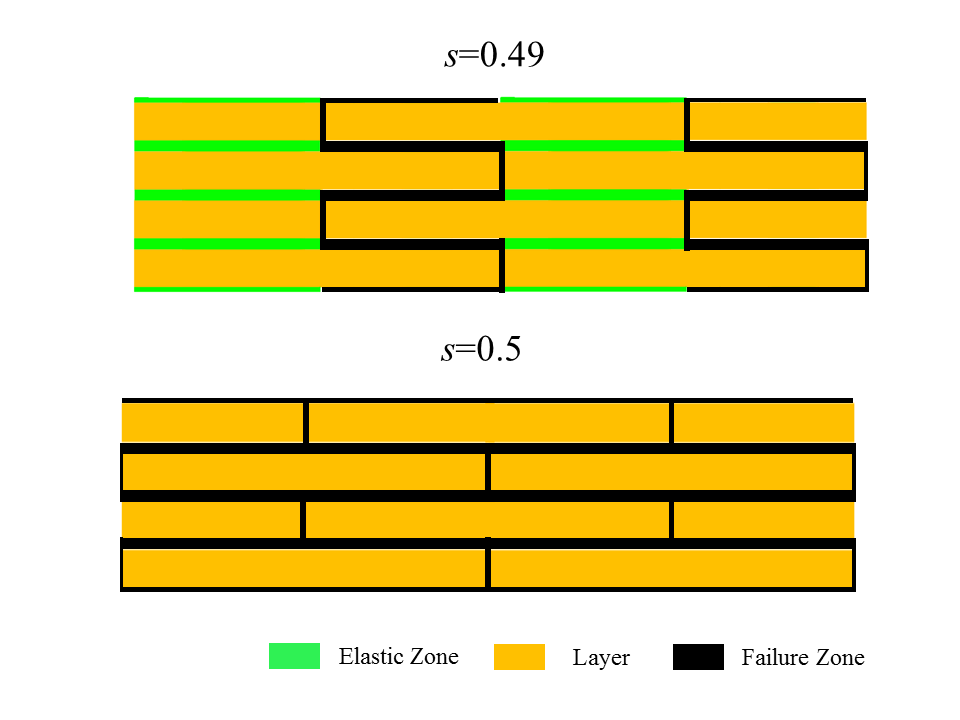


(b)

Figure S4 (a) the stress-strain curve of regularly discontinuous laminated structure without offset ($s=0.5$) and regular discontinuous laminated structure with offset ($s=0.49$), and the corresponding microstructure with (b) single-sided interface failure in the laminated structure with offset and double-sided interface failure in the laminated structure without offset.


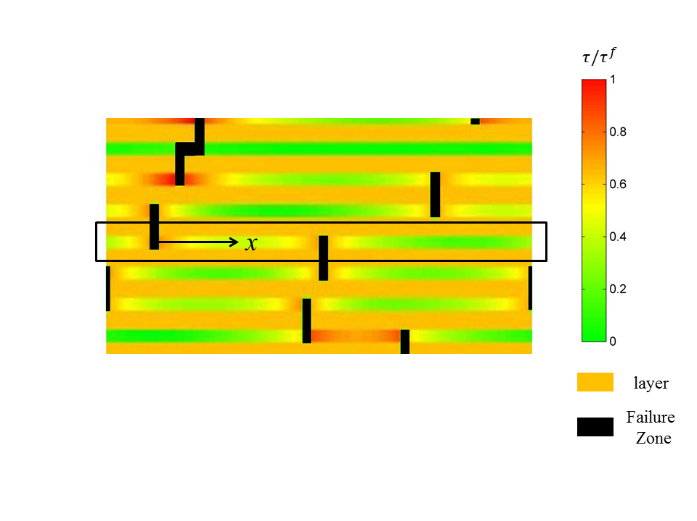

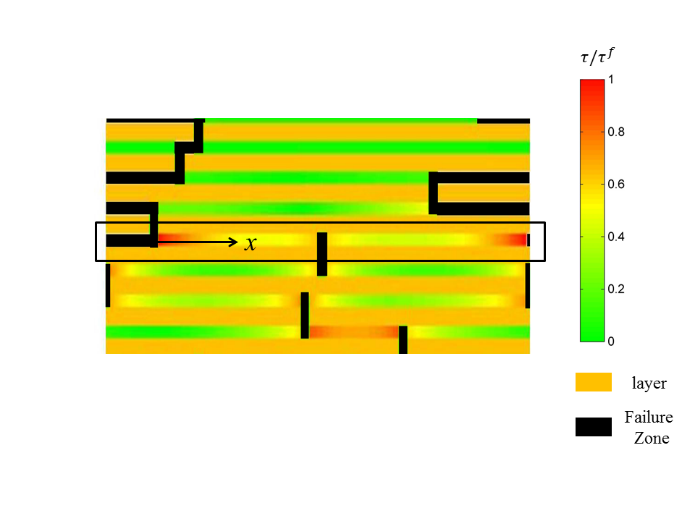


(a) (b)


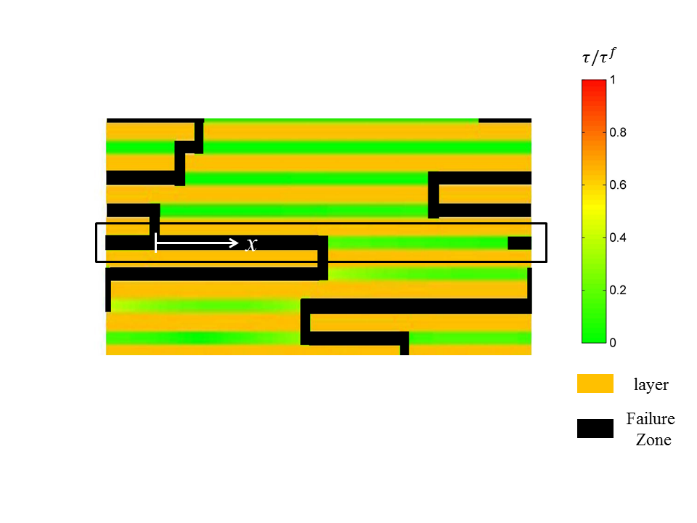


(c) (d)

Figure S5 the part of microstructure of randomly discontinuous laminated structure with $\Delta s=0.18$ and $\bar{l}_{d}/l_{0}=1.85$under different strain (a)$\varepsilon/\varepsilon_{0}=1.04$, (b)$\varepsilon/\varepsilon_{0}=1.05$and (c)$\varepsilon/\varepsilon_{0}=1.14$. In black rectangle, the shear stress distribution on interface in figure (d) shows how progressive failure changes of neighboring shear stress.

**4. Appendix D: Data of laminar materials**

| Composites | Platelet layers | Interlayers | 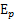  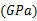 | 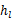  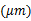 | 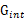  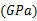 | 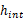  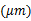 |  | 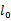  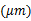 | 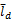^*^  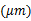 |
| --- | --- | --- | --- | --- | --- | --- | --- | --- | --- |
| **Si_3_N_4_-matrix** |  |  |  |  |  |  |  |  |  |
| BS-5^1^ | Si_3_N_4_ | BN | 220 | 100 | 56.8 | 45 | 0.69 | 132 | 4480 |
| BS-10^1^ |  |  | 220 | 100 | 60.6 | 30 | 0.77 | 104 | 185 |
| BS-50^1^ |  |  | 220 | 100 | 63 | 30 | 0.77 | 106 | 4 |
| Si_3_N_4_/BN^2^ |  |  | 220 | 87 | 56.8 | 25 | 0.78 | 92 | 1760 |
| **Al_2_O_3_-matrix** |  |  |  |  |  |  |  |  |  |
| Al_2_O_3_/Al-Si (25/50μm)^3^ | Al_2_O_3_ | Al-Si | 370 | 25 | 30.4 | 50 | 0.33 | 124 | 426 |
| Al_2_O_3_/Al-Si (10/20μm)^3^ |  | Al-Si | 370 | 10 | 30.4 | 20 | 0.33 | 50 | 18 |
| Al_2_O_3_/SiC^4^ |  | SiC | 370 | 90 | 181.5 | 10 | 0.90 | 42 | 753 |
| Al_2_O_3_ /LaPO_4_^5^ |  | LaPO_4_ | 370 | 100 | 51.2 | 40 | 0.71 | 170 | 508 |
| **Others** |  |  |  |  |  |  |  |  |  |
| SiC/graphite^6^ | SiC | graphite | 450 | 90 | 5.5 | 10 | 0.90 | 270 | 1379 |
| Al_2_O_3_-ZrO_2_/  LaPO_4_^7^ | Al_2_O_3_-  ZrO_2_ | LaPO_4_ | 300 | 25 | 51.2 | 11 | 0.69 | 40 | 1427 |
| Ti_45_Al_40_ (II)^8^ | Ti | Al_3_Ti | 116 | 285 | 56 | 154 | 0.65 | 300 | 410 |
| B_4_C/B_4_C-C^9^ | B_4_C | B_4_C-C | 450 | 2625 | 194 | 90 | 0.97 | 738 | 2000 |

Table 1 the mechanical and geometric properties of some discontinuous laminar materials, and two characterized parameters that are the mean overlap length $\bar{l}_{d}$, and critical length of the platelets $l_{0}$ (red color represents notable double-sided interface failure, and blue color represents single-sided interface failure).

*the lengths of mean overlap lengths of platelet $\bar{l}_{d}$ are described by the longest lengths of interface failure in laminar materials.

**References**

1. Li, X., Zou, L., Ni, H., Reynolds, A. P., Wang, C. A. & Huang, Y. Micro/nanoscale mechanical characterization and in situ observation of cracking of laminated Si3N4/BN composites. *Mat. Sci. Eng. C* **28**, 1501-1508 (2008).
2. Wang, C. A., Huang, Y., Zan, Q., Guo, H. & Cai, S. Biomimetic structure design—a possible approach to change the brittleness of ceramics in nature. *Mat. Sci. Eng. C* **11**, 9-12 (2000).
3. Launey, M. E., Munch, E., Alsem, D. H., Saiz, E., Tomsia, A. P. & Ritchie, R. O. A novel biomimetic approach to the design of high-performance ceramic–metal composites. *J. R. Soc. Interface* **7**, 741-753 (2010).
4. She, J., Inoue, T. & Ueno, K. Multilayer Al_2_O3/SiC ceramics with improved mechanical behavior. *J. Eur. Ceram. Soc.* **20**, 1771-1775 (2000).
5. Tomaszewski, H., Węglarz, H., Wajler, A., Boniecki, M. & Kalinski, D. Multilayer ceramic composites with high failure resistance. *J. Eur. Ceram. Soc.* **27**, 1373-1377 (2007).
6. Vandeperre, L. J. & Van Der Biest, O. O. Graceful failure of laminated ceramic tubes produced by electrophoretic deposition. *J. Eur. Ceram. Soc.* **18**, 1915-1921 (1998).
7. Marshall, D. B., Morgan, P. E. & Housley, R. M. Debonding in multilayered composites of zirconia and LaPO4. *J. Am. Ceram. Soc.* **80**, 1677-1683 (1997).
8. Peng, L. M., Li, H. & Wang, J. H. Processing and mechanical behavior of laminated titanium–titanium tri-aluminide (Ti–Al3Ti) composites. *Mat. Sci. Eng. A* **406**, 309-318 (2005).
9. Orlovskaya, N. *et al*. Boron carbide/boron carbide-carbon nanofibers laminates with weak interfaces. *Nato Sci. Peace Sec. B*, 1-11 (2010).

1. [↑](#footnote-ref-2)
